# Altered Fetal Skeletal Muscle Nutrient Metabolism Following an Adverse *In Utero* Environment and the Modulation of Later Life Insulin Sensitivity

**DOI:** 10.3390/nu7021202

**Published:** 2015-02-12

**Authors:** Kristyn Dunlop, Megan Cedrone, James F. Staples, Timothy R.H. Regnault

**Affiliations:** 1 Department of Physiology and Pharmacology, Western University, London, ON N6A-5C1, Canada; E-Mail: kdunlo@uwo.ca; 2 Department of Biology, Western University, London, ON N6A 5B7, Canada; E-Mails: mcedrone@uwo.ca (M.C.); jfstaple@uwo.ca (J.F.S.); 3 Department of Obstetrics and Gynecology, Western University, London, ON N6H-5W9, Canada; 4 Lawson Health Research Institute, London, ON N6C-2R5, Canada; 5 Children’s Health Research Institute, London, ON N6C-2V5, Canada

**Keywords:** IUGR, hypoxia, obese, insulin sensitivity, skeletal muscle, mitochondria, β-oxidation, fats, acylcarnitine, oxygen

## Abstract

The importance of the *in utero* environment as a contributor to later life metabolic disease has been demonstrated in both human and animal studies. In this review, we consider how disruption of normal fetal growth may impact skeletal muscle metabolic development, ultimately leading to insulin resistance and decreased insulin sensitivity, a key precursor to later life metabolic disease. In cases of intrauterine growth restriction (IUGR) associated with hypoxia, where the fetus fails to reach its full growth potential, low birth weight (LBW) is often the outcome, and early in postnatal life, LBW individuals display modifications in the insulin-signaling pathway, a critical precursor to insulin resistance. In this review, we will present literature detailing the classical development of insulin resistance in IUGR, but also discuss how this impaired development, when challenged with a postnatal Western diet, may potentially contribute to the development of later life insulin resistance. Considering the important role of the skeletal muscle in insulin resistance pathogenesis, understanding the *in utero* programmed origins of skeletal muscle deficiencies in insulin sensitivity and how they may interact with an adverse postnatal environment, is an important step in highlighting potential therapeutic options for LBW offspring born of pregnancies characterized by placental insufficiency.

## 1. Introduction

Recent clinical, epidemiological and animal studies have highlighted an association between an altered, adverse *in utero* environment and growth, and the subsequent propensity of these offspring to develop hallmarks of the metabolic syndrome [1,2,3]. The metabolic syndrome is a cluster of factors indicative of altered metabolism, including hypertension, visceral obesity, glucose intolerance and dyslipidemia, that predispose the patient to the development of comorbidities such as cardiovascular disease, non-alcoholic fatty liver disease and type 2 diabetes [4]. Decreased insulin sensitivity, or insulin resistance, is a critical precursor of the metabolic syndrome, as it is typically evident before other overt symptoms are apparent; and therefore it may represent an early, key step in the pathophysiological progression towards the metabolic syndrome [4].

It has been postulated that the origins of the metabolic syndrome, and insulin resistance specifically, originate during fetal development and early postnatal life [5]. As such, an adverse *in utero* environment has the potential to influence the relative risk of the offspring to the development of aberrant nutrient metabolism in later life, independently of postnatal diet. An adverse *in utero* environment, often characterized by suboptimal nutrient transfer to the fetus, culminates in intrauterine growth restriction (IUGR). IUGR is the endpoint of a continuum of conditions that result in the failure of the fetus to attain its inherent growth potential, which can be diagnosed using ultrasonography during pregnancy [6]. Clinically, IUGR often results in a baby born with a weight or length below the 10th percentile for gestational age [6,7].

The etiology of IUGR is multifactorial, with adverse environmental or genetic and epigenetic factors likely playing a role in the abnormal growth and development of the fetus. One of the most important environmental factors regulating fetal growth, is nutrient delivery to the fetus via placental diffusion and transport [7]. A reduced functional capacity of the placenta, or placental insufficiency, is typically associated with poor placental vascular development, which prevents adequate nutrition and oxygen from reaching the developing fetus, resulting in a hypoxic, nutrient deprived *in utero* environment. Interestingly, it should be noted that, independent of reductions in nutrient supply, hypoxia alone has been shown to have a significant impact on fetal growth, emphasizing hypoxia as a key contributor to impaired fetal growth and potentially IUGR [8].

With exposure to a hypoxic *in utero* environment, the fetus undergoes some key adaptations to ensure survival. A key component of this adaptation in the acute setting has been considered a redistribution of fetal cardiac output towards essential organs such as the brain, heart and adrenal glands, at the expense of other organ systems, including the lungs, kidney, liver and skeletal muscle [9,10,11]. With prolonged hypoxia, animal and human studies have highlighted a redistribution of blood flow toward the brain, as well as increased blood flow towards adrenal glands [12,13,14,15], and it is inferred from this redistribution that the brain continues to receive sufficient perfusion and nutrient supply to maintain relative growth. This brain sparing effect is visible at birth, with the size of the fetal head being larger than that of the abdomen, giving rise to an observable asymmetrical growth restriction. Concurrent with the overall reduction in body weight and brain sparing effect, fetuses exposed to hypoxic *in utero* environments also display a reduced muscle mass compared with normal birth weight offspring [16,17], and are predisposed to altered insulin sensitivity [1,3,5,18]. Furthermore, rodent models of low birth weight have also demonstrated a decrease in skeletal muscle mass [19], similar to the altered muscle to fat ratio observed in older men who were born low birth weight [20]. With this altered development there are metabolic changes to the offspring, resulting in what has been proposed as a “thrifty phenotype” [1,18]. The “thrifty phenotype” encompasses a collection of metabolic adaptations initiated to aid in fetal survival when challenged with nutrient deprivation *in utero* [21]*.* While the “thrifty phenotype” represents the observable phenotype associated with IUGR, theories of fetal programming postulate that altered oxygen and nutrient transfer during critical windows of development, when the fetus is most sensitive to its environment, are associated with permanent alterations in structure and metabolism, and a fixed functional capacity of vital organs in postnatal life [1]. Since the plasticity of the organs *in utero* is lost in postnatal life, adaptations to these metabolic organs initiated *in* *utero* may persist into adulthood, increasing the propensity for these offspring to develop metabolic disease with age [1,22].

Markers of altered fetal growth (including low weight at birth and asymmetric growth) are most widely used as indicators of IUGR or a hypoxic *in utero* environment. However, more subtle adaptations at the physiological level may be the drivers underlying the observable later life phenotypes, such as changes in skeletal muscle metabolic function and anabolic capacity, and overall oxygen consumption. Once the organs have fully developed *in utero,* the IUGR fetus faced with a postnatal environment characterized by nutrient excess, a highly prevalent and easily accessible diet in modern society, may develop long-term adverse metabolic consequences [1,5,18]. Unfortunately, the mechanisms underlying these alterations in the metabolic capacity of the IUGR fetus and their propensity towards the development of later life insulin resistance and the metabolic syndrome remain poorly defined.

## 2. Skeletal Muscle Insulin Signaling and IUGR

Skeletal muscle is the primary location for insulin-stimulated glucose uptake, accounting for up to 70% of whole body glucose disposal [23], and is a key regulator of whole body energy metabolism [24], with other metabolic organs, including liver, adipose tissue and pancreas also involved in the insulin response and pathogenesis of insulin resistance. The primary metabolic objective in the skeletal muscle is production of ATP for contractile purposes; however, skeletal muscle is also responsible for the production and storage of glycogen, an insulin dependent process that provides the cells with glucose for ATP production when circulating levels are low. β-oxidation, a process whereby free fatty acids are broken down to provide muscle with carbon chain substrates, is also important for skeletal muscle ATP production. Since the skeletal muscle is a critical producer of ATP and an important location for glucose and fat metabolism, determining the propensity of skeletal muscle towards developing insulin resistance is a key determinant in the pathophysiological progression towards metabolic syndrome.

Depressed insulin sensitivity, or insulin resistance, is a metabolic state in which peripheral tissues, such as skeletal muscle, are no longer responsive to the anabolic effects of insulin, thereby reducing insulin-stimulated glucose uptake and perpetuating a state of hyperglycemia. Insulin resistance at the level of the skeletal muscle has been associated with a modulation of the serine/threonine phosphorylation status of insulin receptor substrates (IRS) [25,26,27]. A relative increase in the serine phosphorylation of IRS-1, the predominant isoform in skeletal muscle [28], at Ser^307^ reduces its ability to activate or complex with phosphatidylinositol 3-kinase (PI3-kinase). This activation failure impairs downstream phosphorylation of protein kinase B (Akt) at Ser^473^, and Akt Substrate of 160 kDa (AS160) at Thr^642^, ultimately leading to a reduction in glucose uptake into skeletal muscle cells through glucose transporter 4 (GLUT4) transporters [25,27,29].

A hypoxic *in utero* environment, commonly associated with IUGR, is known to negatively influence the fetus during critical periods of development [1,5,18]. Interestingly, IUGR offspring in animal models display improved insulin sensitivity in very early postnatal life, as assessed by intravenous glucose tolerance challenge; however, a shift towards impaired glucose clearance and decreased insulin sensitivity is evident as these offspring age [30,31,32]. The timing of this shift in insulin sensitivity appears to be sex-specific, with insulin action being impaired earlier in males and later in females [30]. These animal studies highlight that sexually dimorphic effects occur with adverse *in utero* environments, and in the case of insulin resistance, studies suggest males appear to be more susceptible to a programmed later life insulin resistance [30,33].

Interestingly however, this sex-specific programming of insulin resistance may not exist in humans. In a mixed cohort of offspring aged 25 who were growth restricted *in utero*, a significantly lower insulin-stimulated glucose uptake was observed compared to controls, as well as a higher plasma insulin concentration, suggesting the development of insulin resistance. Of note, these observations were in conjunction with a normal glucose tolerance challenge, thus representing an early phase in the pathogenesis to insulin resistance and the metabolic syndrome [34]. However, by age 64, men who were born small exhibited a strong link with impaired glucose tolerance and type 2 diabetes [35], while data concerning women born small is not widely available. Therefore, understanding the early steps in the progression towards insulin resistance in the low birth weight population, and any sex-specific effects, is of critical importance for mitigating the risk of these offspring to the development of later life type 2 diabetes.

Animal models of IUGR, as well as some human studies, have also been used in order to identify the molecular changes occurring at the level of the insulin-signaling cascade that may be underlying the reduced insulin sensitivity in this population [29,36]. In early postnatal life, expression of the insulin receptor is increased in low birth weight sheep, as a compensatory mechanism for the low insulin and glucose levels experienced *in utero* [37]. However, the expression of the insulin receptor is no different to normal birth weight controls in skeletal muscle of older low birth weight rodent offspring, highlighting that the later life alterations in insulin sensitivity are likely due to a defect down-stream of the receptor that affects the ability of GLUT4 to translocate to the membrane and take glucose into the cell [36]. Analysis of key intermediates in the insulin signaling cascade has demonstrated sex-specific alterations that may be responsible for altered GLUT4 expression and reduced glucose uptake that is associated with states of insulin resistance [17,29,36,38].

In rodent models of placental insufficiency, young adolescent male offspring show altered GLUT4 transport in conjunction with an increased phosphorylation of IRS-1 [29], which is known to blunt the physiological response to insulin [27] by reducing the coupling efficiency of the insulin receptor and IRS-1 [39]. IRS-1 is also known to complex with PI3-kinase, and in young male low birth weight offspring, reduced expression of the p85 regulatory subunit and p110β catalytic subunit of PI3K has also been observed in rodent, as well as larger animal (e.g., sheep) models of placental insufficiency [31,36,37]. Downstream of IRS-1, the total Akt levels were not altered by insulin infusion in growth restricted males [29,36]; however, phosphorylation of this intermediate was increased by insulin, suggesting a compensatory mechanism may be at play to maintain this physiological response to exogenous insulin [29]. Although Akt’s involvement in the insulin cascade appears to be unaffected, downstream AS160 shows reduced phosphorylation in response to insulin [29], suggesting a functional impairment that impacts GLUT4 translocation to the plasma membrane [40].

In young adolescent female rodent offspring, the phosphorylation status of Akt is higher in the basal state, and insulin infusion is still able to increase the phosphorylation further. Additionally, there are no changes in the phosphorylation of protein kinase C isoforms and an increased phosphorylation of phosphoinositide-dependent kinase (PDK), all of which point to programming of heightened insulin sensitivity [38]. This heightened sensitivity is in accordance with the early improvement observed in sheep models [30], and may represent a protective response initiated to maintain whole body insulin sensitivity at this early age [38]. However, despite this enhanced sensitivity during the growth phase, with age these offspring exhibited a decrease in insulin sensitivity and progression towards an insulin resistant phenotype [41].

Human data has suggested similar molecular alterations are present in young males born low birth weight, including reduced expression of the p85 and p110β subunits of PI3-kinase and reduced skeletal muscle GLUT4 content [16,36]. These alterations occur in conjunction with a blunted phosphorylation of Akt in response to insulin infusion, but with maintenance of glucose tolerance and whole body insulin sensitivity [16,36]. Taken together, changes in the molecular expression of key insulin signaling intermediates in skeletal muscle of low birth weight offspring may precede development of whole body insulin resistance and glucose intolerance, representing an early defect in metabolism that could be indicative of future metabolic disease [36]. However, the mechanisms by which the *in utero* environment may be modulating these changes in insulin sensitivity remain ill defined.

## 3. Fiber Type, Oxygen Consumption and IUGR

Skeletal muscle has a high demand for energy in order to perform its contractile function, with most of its energy requirement being met through oxidative phosphorylation (OXPHOS). While OXPHOS produces greater amounts of adenine triphosphate (ATP) than glycolysis, it is an aerobic process requiring oxygen, conducted within the Krebs cycle and electron transport system of the mitochondria. The density of mitochondria is the primary determinant of oxidative capacity, and this is ultimately set by fiber type and fiber distribution within the skeletal muscle bed. There are a number of different skeletal muscle fiber types distinguished primarily by their oxidative capacity. Type Ia (slow oxidative) fibers are the most mitochondria-rich fibers, and type IIb (fast glycolytic) fibers have the least mitochondria and thus the lowest oxidative capacity. Types IIa and IIx (fast oxidative) fibers have an intermediate number of mitochondria and oxidative capacity [42]. Maximal rates of OXPHOS is dictated by mitochondrial number and consequently directly related to fiber type composition [43,44].

OXPHOS is adversely affected in skeletal muscle of growth-restricted animals [19,45,46], in conjunction with decreased mitochondrial number [19]. These factors, combined with a lowered ATP production are associated with the development of insulin resistance [47,48]. The individual OXPHOS complexes, including ATPase activity, have been studied using respirometry, a technique measuring oxygen consumption using a number of substrates that are metabolized by individual complexes within a closed cell chamber. The rate of oxygen consumption is measured in “states”, where state 1 represents baseline respiration, state 2 follows the addition of malate and glutamate, malate and pyruvate (metabolized as NADH at complex 1) or rotenone and succinate (rotenone inhibiting complex 1 and succinate metabolized at complex 2). State 3 respiration follows the addition of ADP (measuring ATPase activity) and state 4 follows the addition of oligomycin (inhibiting ATPase activity). The ratio of state 3/state 4 respiration rates, termed the respiratory control ratio (RCR), is commonly used to measure mitochondrial dysfunction. Generally, a low RCR is associated with mitochondrial dysfunction as it measures how tightly respiration and phosphorylation are coupled. In rats, IUGR skeletal muscle state 3 was decreased irrespective of substrate used, compared to controls. 

In addition to impaired OXPHOS reported in IUGR rats, reduced fetal glucose oxidation is observed in the heat stress ovine IUGR model [49]. These changes may reflect in the *in utero* determination of skeletal muscle fiber type composition of low birth weight offspring later in life [17,19,50]. In human studies, low birth weight is associated with an increase in type II fibers and no change in type I fibers at 19 years of age [17], and an increased proportion of glycolytic fibers are reported to precede insulin resistance in the *vastus lateralis* of low birth weight males [17]. In rodent studies, IUGR offspring exhibit a lower proportion of type Ia muscle fibers [19] and a shift towards more glycolytic (type IIb) fibers [17], ultimately altering the oxidative capacity and GLUT4 content of the muscle fibers. The results of these adverse *in utero* associated changes however, is not always consistent. For instance, in IUGR piglet studies, the proportion of type I fibers increase in the hind-limb muscles, but the proportion of type IIb were not reported due to methodological difficulties [51,52]. These conflicting conclusions may lie, in part, to species differences, age of fiber collection, but also may be due to the different muscle groups studied.

It is interesting to note that the alterations in skeletal muscle fiber composition and function early in life in low birth weight, IUGR offspring as reported above, appear similar to those observed in later life obese and type 2 diabetic individuals [53,54]. This similarity potentially highlights possible similar mechanisms at play in low birth weight offspring as those at work in patients with a propensity to develop insulin resistance and subsequent type 2 diabetes with age, independent of birth weight and an adverse *in utero* environment. Understanding these similarities will help define the mechanisms underlying altered fiber type distribution and oxygen utilization changes in these various disease states.

## 4. The Impact of IUGR and Later Life Impaired Skeletal Muscle Fat Metabolism upon the Progression of Insulin Resistance

A key traditional factor identified in the development of insulin resistance has been poor diet. The increasing prevalence of a “Western”, or energy-dense, high-fat diet has been implicated as a key-contributing factor in the pathogenesis of insulin resistance, promoting accumulation of fat within the skeletal muscle and impacting mitochondrial function [55,56,57], leading to a diminished mitochondrial fatty acid oxidation capacity [58]. Consumption of this diet generates a surplus of free fatty acids, ultimately leading to ectopic lipid accumulation in non-adipose tissues such as skeletal muscle [59]. Once in the skeletal muscle, excess fatty acids are activated to form their acyl-CoA derivatives, which can be esterified into diacylglycerol (DAG), metabolized into ceramide, or conjugated to acylcarnitine for entry into the mitochondria to undergo β-oxidation [60]. Excess lipids may also be stored in lipid droplets as triacylglycerols, generating a pool of substrates termed intramyocellular triglycerides (IMTG) [56]. IMTG content is known to increase with percentage body fat, and to be elevated in obese or type 2 diabetic individuals. IMTGs are broken down by lipases to undergo oxidation, however disturbances in the rate of breakdown or oxidation may lead to accumulation of toxic lipid intermediates and subsequent insulin resistance [56,61]. The type of triglyceride (saturated *vs.* unsaturated) that make up the skeletal muscle lipid pool is also important, since unsaturated triglycerides may be destined to accumulate as IMTG, whereas saturated triglycerides may be broken down into DAGs [62]. Additionally, a higher proportion of saturated fatty acids within this lipid pool has recently been associated with insulin resistance [62]. Skeletal muscle relies heavily on fatty acid β-oxidation to generate energy, using this method to provide up to 90% of its total energy demand [58]. Therefore, any alterations in fatty acid oxidation may impair skeletal muscle metabolic capacity. Certainly in rodent IUGR studies, muscle oxidative ability is adversely affected in growth-restricted animals [19,45], and as such could set the stage for impaired mitochondrial function when challenged with a postnatal Western diet.

In rodent lipid infusion studies, insulin resistance develops, and increased concentrations of long-chain acyl CoAs have been associated with insulin resistant skeletal muscle [25]. This increased lipid availability has been associated with reduced levels of skeletal muscle β-oxidation [63,64], leading to accumulation of toxic lipid metabolites including DAG [25] and ceramide [65]. While specific changes in long-chain acyl CoA levels have not been reported in IUGR/low birth weight offspring, reductions in enzymes involved in β-oxidation have [66], suggesting that accumulation of long-chain acyl CoAs secondary to a reduction in oxidative capacity may be involved in the pathogenesis of insulin resistance. Accumulation of these lipid metabolites in the skeletal muscle has been associated with increases in stress-induced kinases, such as protein kinase C (PKC)-θ or ε, isoforms known to act upstream of c-Jun N-terminal kinase (JNK) and IκB (inhibitor of NFκB) kinase (IKKβ). Notably, JNK and IKKβ are two central serine/threonine kinases mediating phosphorylation of IRS-1 at Ser^307^, a subsequent reduction in Ser^473^ phosphorylation of Akt, and reduced insulin-stimulated glucose uptake [25,60,65,67], similar to the molecular alterations that have been observed in the insulin signaling pathway in skeletal muscle of the low birth weight population [29,36]. PKC-θ activation itself (translocation from the cytosol to the plasma membrane) has also been reported to occur during a state of lipid overload in skeletal muscle, and may represent an alternative pathway mediating alterations in the serine/threonine phosphorylation status of key insulin signaling intermediates [25,61]. Direct evidence that these changes may be happening in low birth weight IUGR offspring as they age is still lacking, but if occurring, presents a potential pathway where *in utero* induced changes in fatty acid oxidation may play a contributing role to later life insulin resistance when challenged with a postnatal high fat diet.

While the concept of reduced fatty acid oxidation has been held as a corner stone of the development of insulin resistance, new studies now suggest excessive, rather than diminished, fatty acid oxidation in skeletal muscle mitochondria may be the root cause. Evidence for this new concept comes from rodent studies, where animals were fed high-fat diets and subsequently display a metabolic phenotype associated with mitochondrial overload, a situation in which an increase in fatty acid uptake and β-oxidation rate is stimulated, but later steps of metabolism, including the tricarboxylic acid (TCA) cycle or the electron transport chain, remain unaltered [26,58]. This mismatch between subsequent steps of oxidation allows accumulation of acylcarnitine intermediates that are indicative of incomplete oxidation or partial fatty acid degradation [26,58]. Incomplete oxidation occurs during mitochondrial overload because the high rates of β-oxidation generates excessive intermediates that overwhelm the TCA cycle, preventing further oxidation and allowing accumulation of acylcarnitines [26,58]. The recent interest in metabolomics has allowed for profiling of these acylcarnitine intermediates, highlighting a method that allows for further investigation of the relationship between incomplete β-oxidation and the pathogenesis of insulin resistance. High-fat feeding in rodents has been shown to induce a lipid profile high in even-chain acylcarnitines. Accumulation of these even-chain acylcarnitine species ranging in length from C6-C22 indicates that a large proportion of the fatty acids entering the mitochondria are only partially degraded for use as metabolic substrates [26,68]. Of interest, recent evidence has suggested that one even-chain acylcarnitine in particular, L-C14 carnitine, which can accumulate with incomplete β-oxidation, has the ability to activate pro-inflammatory pathways, as well as an induction of JNK which may modulate the serine/threonine phosphorylation status of insulin signaling intermediates, perpetuating a state of insulin resistance [67,69]. Mitochondrial overload has also been observed in rodent models of diabetes, including the Zucker Diabetic Fatty rat, further supporting the idea that incomplete fatty acid oxidation and accumulation of acylcarnitine intermediates may be implicated in the pathogenesis of insulin resistance [26,70]. The impact of high fat feeding in low birth weight IUGR offspring upon acylcarnitine production and mitochondrial overload has not been reported. However, short-term high fat feeding trials in human low birth weight offspring show that low birth weight is associated with a reduced degree of plasticity [71], suggesting that changes to β-oxidation and the TCA cycle, in conjunction with postnatal high fat ingestion, may be different in low birth weight offspring and potentially exacerbated.

The regulation of mitochondrial fatty acid oxidation rate is also an important determinant of the propensity for mitochondrial overload to occur when skeletal muscle is faced with excess free fatty acids. Ingestion of a fatty diet appears to alter the activity of an important family of nuclear transcription factors regulating mitochondrial oxidative capacity, known as the peroxisome proliferator-activated receptors (PPARs). The PPARα isoform is mainly expressed in highly oxidative tissues, including skeletal muscle, liver and heart, with activation of this transcription factor promoting an increase in fatty acid uptake and β-oxidation [58,72]. Interestingly, overexpression of PPARα in skeletal muscle of transgenic mice has been associated with increases of lipid-oxidative genes, yet with the development of glucose intolerance [73], lending support to the notion that over-activation of PPARα in skeletal muscle is detrimental to insulin sensitivity [58]. While it appears that increasing fatty acid oxidation in skeletal muscle during periods of high-fat feeding through activation of PPARα should promote fat clearance by increasing fatty acid oxidation, evidence has suggested that only the genes related to fatty acid oxidation and uptake have been increased, with no changes in the downstream steps of oxidation [68,70]. In addition to detrimental changes in PPARα activity, a chronic high fat diet has been associated with a decrease in skeletal muscle levels of peroxisome proliferator-activated receptor-γ co-activator 1α (PGC1α). PGC1α plays an important role in regulating mitochondrial biogenesis and is a critical component of the PPARα-activated transcriptional machinery [68]. Decreases in PGC1α lead to a reduction in the coupling efficiency between β-oxidation and TCA cycle, preventing the mitochondria from completely oxidizing fatty acids in response to high lipid availability [68]. This decrease in PGC1α levels with consumption of a Western diet would contribute to the mismatch of β-oxidation and TCA cycle activity observed in mitochondrial overload. Recent studies have also reported reduced mRNA levels of PGC1α [74,75] and reduced PGC1α protein levels in the soleus [75] and gastrocnemius [76] muscle of young adolescent low birth weight offspring. Additionally, alterations in the methylation status of the PGC1α promoter have been observed in both rodent [76] and human [77] models of IUGR, highlighting a potential epigenetic link with altered fat storage and metabolism in postnatal life. Therefore, reductions in PGC1α due to consumption of a Western diet, combined with a low birth weight situation could promote further incomplete oxidation and the potential for acylcarnitine intermediates to accumulate in the skeletal muscle and potentiate alterations in the phosphorylation status of insulin signaling molecules through the actions of stress-induced kinases. These emerging studies in the low birth weight population highlight a new area of investigation that is working to better characterize the epigenetic modifications to those genes involved in mitochondrial function and β-oxidation, which is a new and expanding field in understanding the development of insulin resistance in offspring of adverse intrauterine pregnancies.

Collectively, new studies suggest there are alterations in the storage and metabolism of fat in the skeletal muscle of low birth weight offspring. However, the nature of the specific alterations, their degree of plasticity, and their underlying contribution to insulin resistance, when challenged with an adverse postnatal diet as these offspring age, remains to be fully elucidated.

## 5. Conclusions

Infants of an adverse *in utero* environment represent a unique population who appear to be at a greater risk for the development of insulin resistance and subsequent metabolic disease [1,5,18]. Adaptive programmed changes in skeletal muscle metabolic development and function (*i.e.*, changes in insulin signaling, fiber type distribution, oxygen consumption, and oxidative capacity) due to an adverse *in utero* environment, lead to structural and metabolic deficits later in life. Further, and more concerning are recent studies suggesting that these low birth weight offspring are unable lose weight as efficiently as normal birth weight offspring when placed on a calorie restricted diet [19], and that PGC1α deficiencies associated with IUGR appear resistant to exercise intervention [78]. These studies highlight that these unique offspring, while being at a higher risk of developing aspects of metabolic syndrome, may be resistant, or lack plasticity to respond, to current intervention practices. When these infants with programmed alterations in skeletal muscle development are faced with a postnatal environment of nutrient excess, they appear to be at greater risk for aberrant skeletal muscle function. Definitive studies examining the interactive linkages between *in utero* programmed insulin resistance and postnatal environmental challenges, and those investigating the distinct mechanisms underlying the developmental origins of insulin resistance, and a lack of plasticity to later life challenges in low birth weight offspring are needed. With these data, the therapeutic options for this unique population can be better delineated.
